# Extraction and Detection of Avian Influenza Virus From Wetland Sediment Using Enrichment-Based Targeted Resequencing

**DOI:** 10.3389/fvets.2020.00301

**Published:** 2020-05-29

**Authors:** Lauren C. Tindale, Waren Baticados, Jun Duan, Michelle Coombe, Agatha Jassem, Patrick Tang, Miguel Uyaguari-Diaz, Richard Moore, Chelsea Himsworth, William Hsiao, Natalie Prystajecky

**Affiliations:** ^1^Department of Pathology and Laboratory Medicine, University of British Columbia, Vancouver, BC, Canada; ^2^British Columbia Centre for Disease Control Public Health Laboratory, Vancouver, BC, Canada; ^3^School of Population and Public Health, University of British Columbia, Vancouver, BC, Canada; ^4^Animal Health Centre, British Columbia Ministry of Agriculture, Abbotsford, BC, Canada; ^5^Department of Pathology, Sidra Medicine, Doha, Qatar; ^6^Canada's Michael Smith Genome Sciences Centre, BC Cancer, Vancouver, BC, Canada

**Keywords:** avian influenza virus, next generation sequencing, nucleic acid extraction, RT-qPCR, surveillance, sediment, waterfowl

## Abstract

Early virus detection and characterization is key to successful avian influenza virus (AIV) surveillance for the health of humans as well as domestic poultry. We explored a novel sampling approach and molecular strategy using sediment from wetlands and outdoor waterbodies on poultry farms as a population-level proxy of AIV activity in waterfowls. RNA was extracted using the MoBio RNA PowerSoil Total RNA isolation kit with additional chloroform extraction steps to reduce PCR inhibition. AIV matrix protein (MP) gene was detected in 42/345 (12.2%) samples by RT-qPCR; an additional 64 (18.6%) samples showed evidence of amplification below the threshold and were categorized as “suspect positive.” Enrichment-based targeted resequencing (TR) identified AIV sequences in 79/345 (22.9%) samples. TR probes were designed for MP, hemagglutinin (HA), and neuraminidase (NA), however PB2 and PA were also identified. Although RT-qPCR and TR only had fair-moderate agreement, RT-qPCR positivity was predictive of TR-positivity both when using only strictly positive RT-qPCR samples (OR = 11.29) and when coding suspect positives as positive (OR = 7.56). This indicates that RT-qPCR could be used as a screening tool to select samples for virus characterization by TR and that future studies should consider RT-qPCR suspect positives to be positive samples for subsequent resequencing when avoiding false negatives is the priority, for instance in a diagnostic test, and to consider suspect positives to be negative samples when cost efficiency over a large number of samples is the priority, for instance in a surveillance program. A total of 13 HA (H1-7, H9-13, H16) and 9 NA (N1-9) subtypes were identified, with a maximum of 8 HA and 8 NA subtypes detected in a single sample. The optimized RNA extraction and targeted resequencing methods provided increased virus detection and subtyping characterization that could be implemented in an AIV surveillance system.

## Introduction

Avian influenza is a cause of significant morbidity and mortality in domestic poultry around the world. The disease can have substantial economic impacts resulting from production losses, control efforts (including mass depopulations), and restriction on the trade and sale of poultry products (1). Avian influenza virus (AIV) can also cause disease in humans and other domestic and wild animal species. Wild birds are the natural reservoir for AIV (2). They are generally asymptomatic carriers and shed the virus in their excreta, particularly feces (3, 4). Waterfowl spread AIV among geographic locations during their annual migrations, and overlap of migratory flyways facilitates intermixing of birds, AIV reassortment, and the emergence of novel strains (2, 5, 6).

Given the role of wild waterfowl in AIV ecology, they are a primary target for global surveillance efforts. These efforts are primarily centered around the sampling of individual live birds through live trapping, hunter harvest, or collection of birds that have naturally died for other reasons. All of these methodologies are limited in their ability to collect a large and/or representative sample of animals (7). For example, collection of live birds is labor intensive and requires specialized equipment and trained personnel. Hunter harvested animals are often biased by geographic region, timing, and species, depending on when, where, and what hunters are allowed or prefer to hunt. Testing of naturally dead birds (also called passive surveillance) is most economical, but least predictable in terms of the size and composition of the sample.

In the Fraser Valley of British Columbia, the culling of ~17 million (~90%) of the poultry population during the H7N3 outbreak in 2004 also had a cost >400 million Canadian dollars (8–10). Following the devastating H7N3 outbreak, the Canadian Wild Bird AIV surveillance system (based primarily on passive surveillance) was formed to monitor different regions across Canada including BC, Alberta, Saskatchewan, Manitoba, Ontario, Quebec, and Atlantic provinces (8, 11). However, AIV surveillance failed to detect the incursion of the highly pathogenic H5N2 virus in the Fraser Valley in 2014, which resulted in the depopulation of 245,600 birds (12). This detection failure, in combination with the overall low number of birds and low rate of AIV detection for the surveillance system indicated the need for an alternative surveillance strategy.

There is a growing interest in using environmental samples for AIV surveillance, including feces, water and wetland sediments. Among these samples, sediment is theoretically ideal as it may contain contributions from multiple birds and is more concentrated than water. Indeed, previous studies have successfully identified AIV in wetland sediment (4, 6, 13). Wetlands can be a site of significant waterfowl density as they provide important areas of refuge during migration (5). Since wild migratory waterfowl naturally harbor and excrete large amounts of AIV in their feces for prolonged periods (6–28 days) (3, 4), wetlands may contain a diversity of AIVs from multiple avian hosts. This makes wetlands strategic areas for sediment-based AIV surveillance.

While sediment sampling has the benefit of being simple and inexpensive, viral rarity and degradation, as well as the biotic and abiotic complexity of these samples can challenge traditional diagnostic methodologies, such as virus culture and PCR. Soil matrices contain polyphenols, heavy metals, and humic substances: polyphenols cross-link with nucleic acids, heavy metals reduce the specificity of primers, and humic substances interact with DNA polymerase and template DNA (14). These compounds need to be removed sufficiently during extraction so that they do not impact downstream PCR applications; humic substance in particular have been shown to cause inhibition even at low concentrations (15). As well, estimates of ~50,000 operational taxonomic units per gram of soil sample (16, 17) indicate that background species not only outnumber the virus target but also promote sample degradation by nucleases (6, 18). In addition, exposure to physical factors, such as high temperature, strong ultraviolet light, and extreme pH and salinities, hastens viral particle and RNA degradation in the environment (19–21). These challenges of viral rarity, degradation, and the presence of a large metagenomic background can be mitigated using alternative strategies, such as the combination of target enrichment during library preparation and next-generation sequencing (NGS). Enrichment hybridization capture has been widely used to detect low abundance targets including circulating tumor DNA (22), viral integration sites from formalin-fixed paraffin-embedded tumor tissue (23), and to improve on-target rate for sequencing pathogens, such as norovirus out of a complex matrix (24). In the present study we evaluate new laboratory methods for AIV detection and characterization from sediments which enables an alternative strategy for AIV surveillance to complement wild bird sample testing.

## Materials and Methods

### Wetland and Farm Sample Collection

Fifteen wetland areas ranging from <1 to 280 hectares were selected in the Fraser Valley of British Columbia (BC), based on the diversity and abundance of migrating birds in the area based on a citizen-science bird database (www.ebird.org). Five sampling sites per wetland area were chosen and four 50 mL subsamples were collected per site (*n* = 300), with subsamples ~1 m apart from each other and in from the shore. Collection was performed by walking 1–2 m into the water and collecting the superficial layer of submerged sediment into a sterile 50 mL conical falcon tube (25). Samples were transported to the lab and frozen without preservatives at −80°C within 4–8 h of collection (26). Additionally, a 200 mL water sample was collected from each sampling site (*n* = 75) and analyzed for total coliforms and *Escherichia coli* using the Colilert-24 test kit (IDEXX, Maine, USA). It was postulated that higher counts of total coliform and *E. coli* (fecal-indicator bacteria) would suggest higher bird fecal contamination of wetland sediments and therefore an increased likelihood of isolating AIV RNA from the sediment. Sample collection was conducted between January 19 and February 13, 2015 during the AIV outbreak. As well, the Canadian Food Inspection Agency provided 45 sediment samples that were obtained from waterbodies located on poultry farms in the Fraser Valley where the H5N2 virus outbreak strain was detected during the 2014/15 outbreak.

### RNA Extraction

RNA extraction was performed using the RNA PowerSoil Total RNA isolation kit (MoBio Laboratories, Carlsbad, CA, USA) according to manufacturer's recommendations. A chloroform (Sigma-Aldrich Inc., MO, USA) extraction step (27) was added after the phenol:chloroform:isoamyl alcohol (pH 6.5–8.0) (Sigma-Aldrich Inc., MO, USA) extraction step to remove PCR inhibitors. The supernatants were mixed with equal volumes of chloroform followed by centrifugation and repeated once before continuing with the RNA PowerSoil Total RNA isolation protocol. RNA pellets were eluted in 30 μl of RNase-free water, RNA concentrations were quantified using Qubit® RNA HS Assay Kit (Invitrogen by Life Technologies, OR, USA) to assess extraction success, and RNA extracts were stored at −80°C.

### AIV RT-qPCR Analysis

AIV was detected in the samples by real-time reverse transcription polymerase chain reaction (RT-qPCR) targeting the matrix protein (MP) gene segment of AIV using M52C and M253R primers, M96C probes (28), and the AgPath-ID™ One-Step RT-qPCR kit (Ambion, Applied Biosystems® by Life Technologies, NY, USA). The final reaction volume of 25 μl consisted of 2 μl total RNA (1:10 dilution), 1 μl of each primer (10 μM), 0.3 μl of probe (10 μM), 12.5 μl 2× RT-qPCR buffer, 1 μl of 25× RT-qPCR enzyme mix and 7.2 μl of nuclease-free water. The MP gene sequence amplification was performed using the 7500 Fast Real-Time PCR System (Applied Biosystems®, Life Technologies, NY, USA) with the following cycling conditions: 10 min at 45°C, 10 min at 95°C, followed by 40 cycles of 15 s at 95°C and 45 s at 60°C. Additional AIV H5 and H7 specific RT-qPCR assays were performed on all samples (29). The PCR amplicon of select AIV positive samples were run on a 1.8% agarose gel to confirm the target amplicon size.

### Evaluation of PCR Inhibitors in RNA Samples

To assess if PCR inhibitors were present in RNA extracts, an inhibition study was carried out on a subset of 45 randomly selected wetland RNA samples using spiked in RNA that would be unique to the sample. A known quantity of West Nile virus (WNV, HNY1999) RNA (Asuragen Inc., TX USA) was tested neat (WNV neat) and also mixed with the soil RNA extract (WNV +soil) and its amplification was evaluated. Inhibition was expressed as delta Ct (ΔCt), which was the absolute value of the difference between the Ct values in the WNV neat and WNV +soil RT-qPCR assays. 1 μl of heat-released (75°C for 3 min) WNV RNA (50,000 copies/μl) was mixed with 1 μl undiluted extracted RNA sample in a 25 μl total reaction volume. The RT-qPCR assay was performed in triplicate using previously reported WNV primers and probes (30) with the same polymerase and cycling conditions as the MP gene RT-qPCR.

### Probe-Based Targeted Resequencing and Bioinformatics Analysis

UniPrep universal library preparation kit was used for library construction. RNA from samples was reversed transcribed into cDNA and dual-indexed Illumina-compatible libraries were constructed.

Targeted resequencing (TR) allows for the selective sequencing of regions of interest through hybridization to biotinylated probes, followed by isolation by magnetic pulldown. TR of the RNA extracts was performed using the Avian Influenza Environmental ONETest assay (Fusion Genomics Corp., Burnaby, CA). Target enrichment of AIV MP, hemagglutinin (HA), and neuraminidase (NA) segments were conducted using Fusion Genomics Corp.'s proprietary QUANTUMrobes (patent-pending) followed by in-solution hybrid capture. Specifically, the biotinylated oligonucleotides probes designed to match MP, HA, and NA are hybridized with the sequencing library and captured by streptavidin-coated magnetic beads, thereby enriching the library for the regions of interest. Probes were designed to be tiled across MP, HA, and NA sequences enabling them to hybridize across the cDNA molecule. It is important to note that once the probe is hybridized to the cDNA molecule, the entire molecule can be captured for sequencing. Libraries were sequenced on the Illumina NextSeq 550 (2 × 150 base pair paired-end reads) (Illumina, CA, USA).

Identification and characterization of AIV subtypes in the samples were done using an in-house-developed bioinformatics analysis pipeline (https://github.com/duanjunhyq/AIV_seeker) as previously described (25). All sequence data were deposited into the NCBI sequence read archive PRJNA353856 (ncbi.nlm.nih.gov/sra).

### H5N2 Control Sequence Coverage Analysis

To determine the evenness of coverage for the HA and NA genes, sequencing depth from H5N2 positive controls was examined separately. H5 and N2 reads obtained from these controls were aligned against reference sequences (H5 GenBank accession number: KP307957; N2 GenBank accession number: KP307959) using Bowtie (31). The alignment results were sorted using SAMtools (32) and sequence coverages were calculated using BEDTools.

### Data Analysis

Statistical analysis was conducted in R 3.5.1 (33). Sequencing and RT-qPCR concordance was calculated using Cohen's Kappa. Logistic regression was used to determine whether total coliforms or *E. coli* counts were predictive of RT-qPCR MP gene positivity, and if RT-qPCR MP gene positivity or Ct value was predictive of targeted sequencing positivity as follows: *y* ~ *independent variable* (function = glm, family = binomial, link = logit).

## Results

The MP gene RT-qPCR analysis found that 12.2% (42/345) of the sediment samples were positive for the AIV MP gene (Table S1); these positives were distributed throughout the collection period (Figure S1). Ct values for the positive samples ranged from 29.32 to 40.00 cycles. An additional 64 (18.6%) samples showed exponential amplification but did not reach the set fluorescence threshold value of 0.1 (relative fluorescence units) before 40 cycles. These were considered suspect positives. The RT-qPCR targeting avian H5 and H7 were negative for all samples. PCR inhibition as measured in ΔCt (tested in a subset of samples (*n* = 45) by spiking samples with WNV RNA and testing its amplification in the presence of the soil extract) was between ΔCt = 0–0.99 in 36/45 samples (80%), between ΔCt = 1.0–1.49 in 7/45 samples (16%), and between 1.5–2.99 in 2/45 samples (4%), with an overall average ΔCt of 0.70 (SD = 0.52). A shift in Ct value of 0.71 would have minimal impact on assay sensitivity/limit of detection; this would be the equivalent of a 2.3-fold dilution.

**Figure 1:**
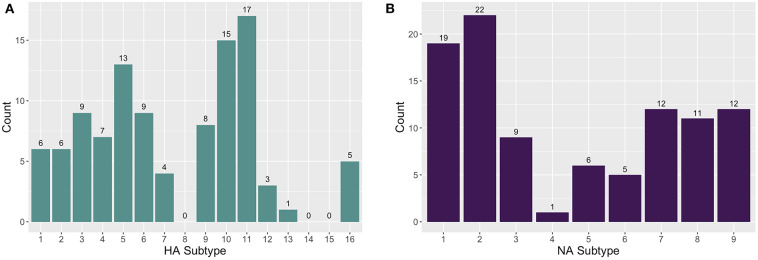
The distribution of **(A)** hemagglutinin (HA) and **(B)** neuraminidase (NA) subtypes found in farm and wetland sediment samples.

The enrichment-based targeted resequencing (TR) identified AIV reads in 79/345 (22.9%) of the RNA extracts, 71 of which contained an HA and/or NA segment. Specifically, 50/79 (63.3%) sequence positive samples contained an HA segment, 52/79 (65.82%) contained an NA segment, and 26/79 (32.9%) contained an MP segment. Additionally, although probes were not designed for other AIV gene segments, 14/79 (17.7%) samples contained a PB2 (polymerase basic protein 2) segment, and 2/79 (2.53%) contained a PA (polymerase acidic protein) segment. The AIV gene segments contain conserved regions at the 5′ and 3′ ends and the probes that are designed for these end regions are likely non-specific between gene segments. As well, non-specific pull-down is common in TR methods (34), especially in cases where the target is in very low abundance in a complex matrix. A maximum number of eight HA and eight NA subtypes were detected in one wetland sample. A total of 13 HA (H1-7, H9-13, H16) and nine NA (N1-9) subtypes in sediment RNA extracts across different wetland and farm sites were identified and characterized (Figure 1). The most commonly sequenced subtypes in the extracted RNA wetland samples were H11 (4.93%; 17/345), H10 (4.35%; 15/345), H5 (3.77%; 13/345), and H3 (2.61%; 9/345) for HA, and N2 (6.38%; 22/345), N1 (5.51%; 19/345), N7 (3.48%; 12/345), and N9 (3.48%; 12/345) for NA (Table S1).

**Figure 2:**
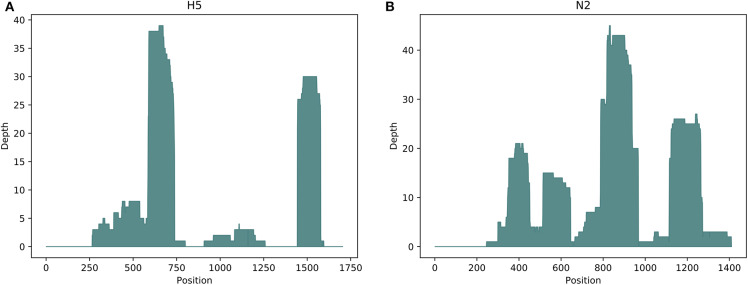
Targeted resequencing depth of coverage for **(A)** H5 and **(B)** N2 reference sequences in H5N2 positive controls. The x-axes show the base position for the H5 and N2 reference sequences. Missingness of reads covering portions of the reference sequence indicates parts of the sequence that were not captured by the probes and sequenced.

The TR depth of coverage for H5 and N2 sequence reads from positive controls is shown in Figure 2. Zero read coverage on portions of the reference sequence indicates parts of the sequence that were either not captured by the probes and sequenced, or due to degraded RNA molecules. The rectangular peaks of good coverage with interspersing areas of low or no coverage, however, suggest that the gaps in coverage are likely due to probe bias. Ideally, if the probes were successfully designed and tiled uniformly across the H5 and N2 sequences, then the probes would bind across the molecule, resulting in the capture and sequencing of entire the gene segment.

The concordance between the AIV MP gene RT-qPCR assay and TR is shown in Table 1. TR and RT-qPCR with suspect positives coded as positive were in agreement 76.5% of the time with kappa = 0.406 (*p* = 1.35E-14), which is categorized as moderate concordance per Altman (35, 36). TR and RT-qPCR with suspect positives coded as negative were in agreement 81.7% of the time with kappa = 0.381 (*p* = 3.06E-14), which is categorized as fair concordance. MP gene RT-qPCR positivity was significantly associated with TR positivity both with suspect positives coded as positive (OR = 7.56, 95% CI = 4.35–13.1) and coded as negative (OR = 11.29, 95% CI = 5.49–23.2). Ct values were predictive of sequence positivity (OR = 0.67, 95% CI = 0.50–0.91, *n* = 42); for every one unit increase in Ct value the odds of getting a TR positive sample decreased by 33%. Furthermore, there was evidence that *E. coli* counts were significantly associated with RT-qPCR positivity, however the odds ratio was very small. With suspect positives coded as negative and positive, respectively, a one unit increase in *E. coli* counts lead to a 1.0063-fold (95% CI = 1.0015–1.0112) and 1.0061-fold (95% CI = 1.0002–1.0119) increase in the odds of a sample being MP gene RT-qPCR positive. There was no evidence of an association between total coliform counts and RT-qPCR positivity.

**Table 1:** Data concordance between avian influenza virus (AIV) matrix protein gene RT-qPCR and target capture resequencing.

**AIV matrix protein gene RT-qPCR (*n* = 345)**	**Target capture resequencing (*****n*** **= 345)**		**Row total**
	**Positive**	**Negative**	
Positive	29	13	42
Suspect positive	23	41	64
Negative	27	212	239
Column total	79	266	345

## Discussion

In this study, we demonstrate that AIV RNA can be successfully extracted from environmental sediment samples, detected using RT-qPCR and characterized using AIV-specific probes for targeted resequencing [clade analysis detailed in Himsworth et al. (25)].

The presence of fulvic and humic acids in wetland sediments can present a challenge as these substances can inhibit the performance of molecular assays, such as PCR; it is therefore essential to optimize extraction methods to reduce their occurrence in RNA extracts (37, 38). Initially, we attempted to use the unmodified RNA PowerSoil Total RNA kit. However, this kit yielded RNA extracts with light to dark brown discoloration, suggesting that humic acids may not have been sufficiently removed (39), and no AIV RNA could be amplified with the MP gene RT-qPCR assay. The addition of PCR enhancers, such as bovine serum albumin (BSA), dimethyl sulfoxide (DMSO) and commercial enhancers did not ameliorate PCR inhibition in these samples. Subsequently, the RNA PowerSoil Total RNA isolation protocol was modified to include the chloroform extraction steps after phenol:chloroform:isoamyl alcohol extraction to further remove PCR inhibitors. The spiking experiments showed that this method is not impacted by PCR inhibitors; either that they were not present or that our testing method minimized any impacts of inhibitors present in the soil using a robust extraction system that included a phenol:chloroform:isoamyl step, as well as the use of PCR additives. Despite the success of the protocol, a chemical fume hood is required when working with phenol:chloroform:isoamyl alcohol, which may be problematic in some laboratory settings; we are therefore exploring alternative protocols and commercially available kits to use in a wider surveillance system.

Given that RT-qPCR is less expensive and more easily accessible than TR, it is preferable to screen sediment samples by RT-qPCR and analyze only RT-qPCR-positive samples by TR. RT-qPCR and TR results showed fair to moderate concordance. RT-qPCR and TR samples had an agreement of 76.5% with kappa = 0.406 and 81.7% with kappa = 0.381 with suspect positives coded as positive and negative, respectively. RT-qPCR positivity was found to be a significant predictor of TR positivity regardless whether the suspect positives were coded as positives or negatives, although the odds ratio was higher when suspect positives were coded as negative (11.29 compared to 7.56). Lower Ct values were also predictive of TR positivity (OR = 0.67). We suggest that future studies consider RT-qPCR suspect positives to be positive samples for subsequent resequencing when avoiding false negatives is the priority, for instance in a diagnostic test, and to consider suspect positives to be negative samples when cost efficiency over a large number of samples is the priority, for instance in a surveillance program.

Disparity between RT-qPCR and TR positive results may be due to sample heterogeneity or state of degradation. However, it is unlikely that there are many false positives among the TR-positive samples, given the conservative pipeline used for QC and filtering during bioinformatics analysis and that the rate of TR positivity (22.9%) is corroborated by the RT-qPCR results, falling in between the RT-qPCR positivity rates including or excluding the suspect positives (30.7% to 12.2%). Furthermore, we suggest that TR is more sensitive for the detection of AIV RNA compared to RT-qPCR, which could be attributable to a number of factors: (1) soil inhibitors or too much metagenomic non-target nucleic acids could generate a false negative in the RT-qPCR assay (40), (2) the subset of AIV RNA within a sample may not contain the MP gene segment that the RT-qPCR assay is designed to detect, and (3) the positive target capture resequencing result could therefore be attributed to the probes being tiled across HA, NA and MP sequences, which had a better chance of picking up viral RNA in the wetland sample for detection. The RT-qPCR and TR disparity is not surprising given that AIV RNA extracted from environmental samples may be fragmented or degraded and may incidentally not contain a sufficient number of full-length MP gene targets. That being said, there were RT-qPCR-positive samples that were TR-negative. This may be attributable to bias introduced by the commercial probes used during sample enrichment. The varying probe sequence depth of coverage across H5 and N2 positive control reference sequences support this claim; however, because of the proprietary commercial probe sequences used, we were unable distinguish the problem of probe bias from RNA degradation. As probe design may be a key factor leading to negative resequencing data, we are developing our own in-house designed probes for future studies.

Another possibility is that the AIV RNA targets were too degraded for NGS sequencing. Rigorous AIV RNA extraction methods of the complex soil matrix as well as exposure to environmental factors, such as RNA degrading components (nucleases) may reduce the RNA sample integrity by degrading or fragmenting the RNA so that the probes are unable to bind. Additionally, the presence of excessive amounts of non-target RNA could have overwhelmed the limited number of AIV RNA segments (“crowding effect”) and blocked the formation of probe-AIV target segment complexes. The unbound target sequences would subsequently be discarded during washing steps and lead to negative results after TR analysis.

Overall, despite the fact that TR is more sensitive than RT-qPCR due to the ability of the probes to capture HA, NA, and MP, compared to a single target of the MP gene segment via RT-qPCR, RT-qPCR positivity and particularly positive samples with low Ct values were predictive of TR-positivity, indicating that RT-qPCR could be used as a screening tool to select samples for virus characterization by TR. Using the TR approach, 13 HA (H1-7, H9-13, H16) and nine NA (N1-9) subtypes were sequenced from the wetland sediment samples. Among RT-qPCR-positive samples, RT-qPCR for AIV H5 and H7 was negative in all samples, however, using TR, H5 and H7 were identified in 14 and 4 samples, respectively. The failure of AIV H5 and H7 RT-qPCR may have arisen from primer mismatches as a consequence of the high mutation rate of HA genes (6, 28). TR is able to overcome the limitation of primer mismatches because the samples are captured using a large pool of probes designed to cover the diversity exhibited in AIV. AIV may still mutate beyond the scope of the designed probe library eventually, but this will occur far more infrequent than when using traditional PCR primers. Moreover, TR can be used when RNA degradation makes RT-qPCR amplification unreliable.

Using an RT-qPCR assay, 12.2% (42/345) of the RNA extracts were positive for the AIV MP gene; an additional 18.5% (64/345) were suspect positive (Table S1). The AIV positivity rate of sediment samples by RT-qPCR and TR (22.9%; 79/345) is comparable to the rate reported in live wild birds (6–37%) and higher than the dead-bird (non-detection to 5%) surveillance programs in Canada (8, 11, 29). However, it is of note that the national surveillance program in 2014/2015 (i.e., at the time of the H5N2 HPAI outbreak) did not detect AIV in any wild birds in BC. This is the timeframe during which the sediment samples were collected, suggesting that sediment approach was more sensitive than traditional passive surveillance. More importantly, in current bird testing each sample only represents a single bird. PCR methods detect the presence of the MP gene and subsequent PCR testing of positive samples is for specific HA subtypes which may or may not be detected due to primer mismatches. In addition to the fact that it is easier, more cost effective, and requires less skill to collect sediment than it does to trap and swab birds, the clear advantage of the TR method is that we are able to test sediment samples that represent a population of birds and because we are capturing AIV with a large pool of probes, we are able to detect a much wider diversity of subtypes including multiple subtypes within the same sample. One limitation to the sampling strategy employed in this study is that we cannot grow AIV from positive sediment samples, which is commonly performed during bird surveillance. As such, the method described in this paper may be best suited to complement existing surveillance programs.

These data show that it is possible to detect and characterize AIV in wetland sediments using a combination of molecular methods. Our optimized RNA extraction method generated RNA extracts suitable for RT-qPCR and TR/NGS. The NGS results confirmed that wetlands contain a wide range of HA and NA subtypes, including multiple subtypes per sample. This suggests that genomics analysis of wetland sediment could complement existing AIV surveillance systems and could, in the future, be used to monitor and mitigate the threat of AIV outbreaks in poultry and other species.

## Data Availability Statement

The datasets generated for this study can be found in the National Center for Biotechnology Information BioProject database (PRJNA353856).

## Author Contributions

NP, WH, CH, and PT: conceptualization and funding acquisition. WB, JD, PT, MU-D, RM, MC, AJ, WH, NP, and CH: methodology. JD: software. LT, JD, and MC: formal analysis. WB and MC: investigation. LT and WB: writing—original draft preparation. NP, WH, and CH: supervision. All authors: writing—review and editing.

## Conflict of Interest

The authors declare that the research was conducted in the absence of any commercial or financial relationships that could be construed as a potential conflict of interest.

## Supplementary Material

Figure S1 Count of samples by collection date that were found to be negative, suspect positive, or positive using RT-qPCR for the MP gene.
