# Supplementary figures and images for: Extraction and Detection of Avian Influenza Virus From Wetland Sediment Using Enrichment-Based Targeted Resequencing
Source: Front Vet Sci. 2020 May 29;7:301. doi: 10.3389/fvets.2020.00301 (PMC7273442; doi:10.3389/fvets.2020.00301)

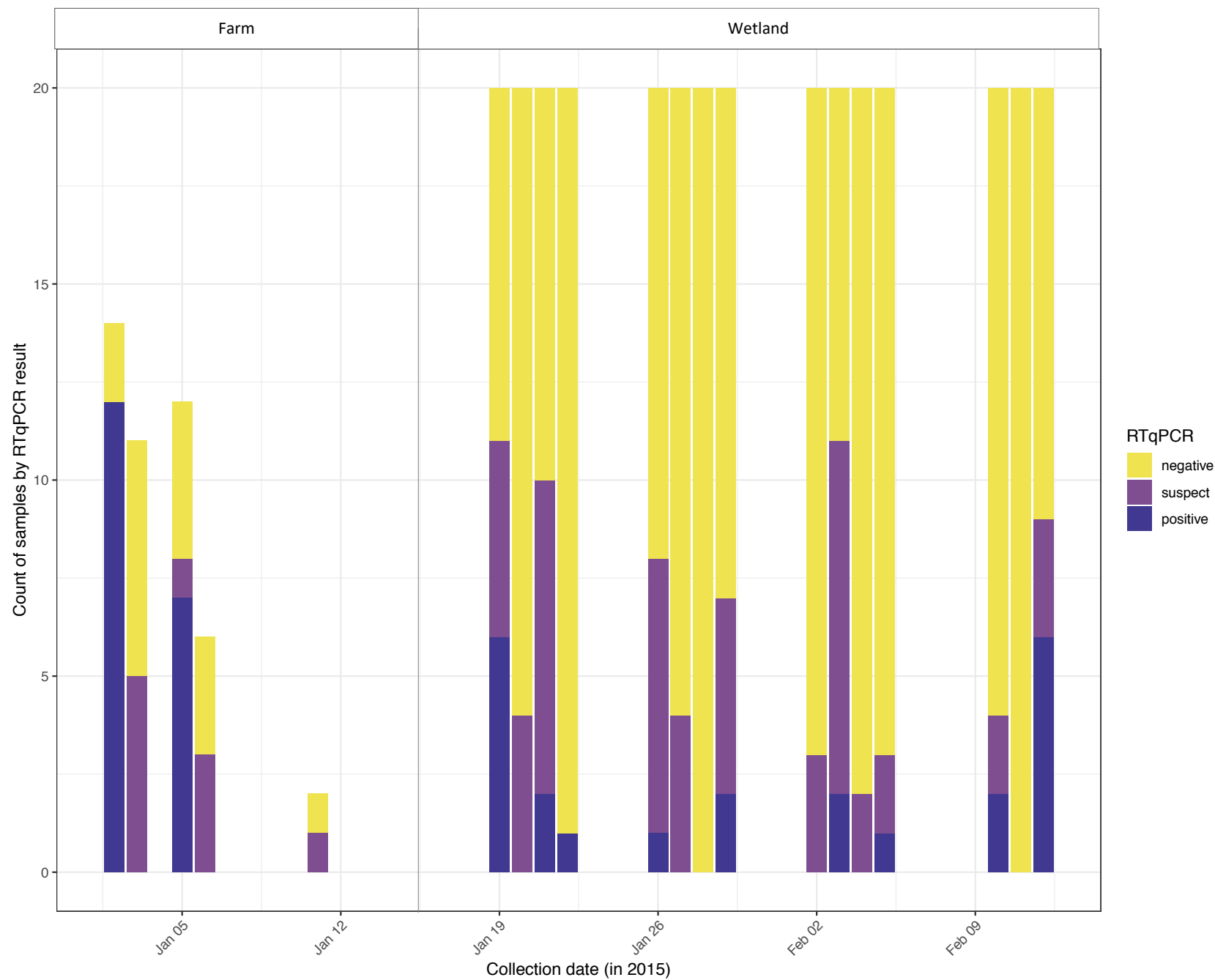

Supplement: Figure S1 — Count of samples by collection date that were found to be negative, suspect positive, or positive using RT-qPCR for the MP gene. [file Image_1.PDF]
